# Structural Determinants of Cost‐Related Healthcare Delay Among Refugee Newcomers in the United States: Evidence From the Annual Survey of Refugees, 2020–2022

**DOI:** 10.1111/1475-6773.70184

**Published:** 2026-11-13

**Authors:** Hyojin Im, Isok Kim, Wooksoo Kim

**Affiliations:** ^1^ School of Social Work, Virginia Commonwealth University Richmond Virginia USA; ^2^ School of Social Work, University at Buffalo Buffalo New York USA

**Keywords:** delayed care, health equity, healthcare access, insurance continuity, refugees, resettlement, structural barriers

## Abstract

**Objective:**

To examine multilevel correlates of cost‐related healthcare delay among refugee newcomers and their families in the United States.

**Study Setting and Design:**

We analyzed pooled cross‐sectional data from the 2020 to 2022 Annual Survey of Refugees, guided by an integrated Andersen‐Hynie framework. Survey‐weighted least absolute shrinkage and selection operator (LASSO) regression selected variables for weighted logistic regression, with region, and survey cycle retained throughout.

**Data Sources and Analytic Sample:**

Public‐use data comprised 4333 adult household respondents with an observed outcome; 3129 had complete candidate‐variable data. Multilingual telephone interviews were conducted in 2021–2023.

**Principal Findings:**

The weighted prevalence of reported cost‐related delay for oneself or a family member was 17.8%. Noncost healthcare barriers and discrimination were associated with increases of 12.7 percentage points (95% confidence interval, 8.8–16.7) and 9.4 percentage points (5.6–13.2), respectively, in adjusted delay probability. Each additional insured month was associated with a 1.1‐percentage‐point decrease (0.6–1.5). Regional differences were not clearly supported after adjustment. Key associations persisted in sensitivity analyses.

**Conclusions:**

Noncost healthcare barriers, discrimination, and shorter insurance coverage were associated with cost‐related delays. The findings support attention to coverage continuity, navigation support, and equitable treatment in healthcare services.

## Keypoints

- What Is Known on This Topic
  ○ Refugees frequently delay needed healthcare due to cost, particularly, during transitions from initial resettlement coverage to longer‐term insurance programs.
  ○ Language barriers, administrative complexity, and uncertainty about costs consistently limit timely care for immigrant and refugee populations.
  ○ Existing research largely examines these barriers in isolation, with limited evidence on how structural, social, and health factors interact to shape delays.
- What This Study Found
  ○ Noncost healthcare barriers and discrimination were associated with more reported delays, while longer insurance coverage was associated with fewer delays.
  ○ Adjusted regional differences were uncertain. Latino/Hispanic subgroup estimates varied but were too imprecise to identify a consistently disadvantaged group.
  ○ Coverage continuity and practical assistance obtaining care warrant attention in refugee health services; the effects of subsequent policy changes require further evaluation.

## 1 Introduction

Delayed access to needed healthcare remains a persistent public health challenge in the United States and is associated with worsening health conditions [1, 2] and premature mortality [3, 4]. National surveys document substantial cost‐related delays [5, 6], and research among adults with diabetes identifies persistent differences by insurance status [7]. Within a complex and fragmented US healthcare system, cost‐related delay is an important phenomenon to examine, especially for vulnerable populations such as refugee newcomers.

These barriers are particularly salient for refugee newcomers, who frequently encounter layered social, economic, and institutional constraints during resettlement. The United States has resettled more than 3.3 million refugees since 1980, when Congress passed the Refugee Act [8]. Refugee Medical Assistance (RMA) provides time‐limited coverage for eligible refugees who do not qualify for Medicaid [9]. As refugee newcomers transition out of this initial assistance period, many face challenging situations of coverage instability, cost uncertainty, and disrupted access to care [10].

There are a number of challenges associated with accessing healthcare for refugee newcomers, such as language and communication barriers [11] and unfamiliarity with the US healthcare system and how to navigate it [12, 13]. While interpretation services and navigation support can mitigate some obstacles, the financial aspects associated with using healthcare remain a pervasive deterrent to timely care. For families unfamiliar with the relationships between providers, insurers, and public assistance programs, these difficulties can make the decision to seek care particularly uncertain.

Despite growing recognition of these challenges, existing research has tended to examine individual, structural, and contextual determinants of healthcare access in isolation. Less is known about how these multilevel factors jointly shape cost‐related delays among refugee newcomers, particularly during the critical period of early resettlement and insurance transition. Addressing this gap is essential for informing equitable health policy, strengthening safety‐net systems, and supporting integration for newcomer refugees.

### 1.1 Conceptual Model: Integrated Andersen‐Hynie Conceptual Framework

In this study, we developed an integrated Andersen–Hynie conceptual framework by combining Andersen's Behavioral Model and Hynie's Holistic Integration Model to examine multilevel determinants of cost‐related healthcare delay among refugee newcomers. Andersen's Behavioral Model [14, 15] conceptualizes healthcare utilization as a function of individual and contextual determinants that shape both access and behavior. It distinguishes three primary domains: predisposing factors (demographic, social, and attitudinal characteristics that influence health‐seeking tendencies), enabling factors (resources and conditions that facilitate or constrain access to care), and need factors (perceived or evaluated health conditions that motivate healthcare use). In later revisions, Andersen emphasized that contextual characteristics, such as community norms, service systems, and structural inequalities, further shape these determinants and reflect how healthcare utilization depends not only on illness and perceived need but also on the social and structural resources available to individuals within their environments [14, 16]. Meanwhile, the Holistic Integration Model [17, 18] complements this framework by conceptualizing integration as a dynamic, multidimensional process encompassing social, interactional, and subjective domains embedded within broader socioeconomic and institutional contexts. Successful adaptation of newcomer refugees and immigrants depends on the interconnections between objective conditions (e.g., employment, language proficiency, and community support) and subjective experiences (e.g., belonging, safety, and trust). By centering both relational and structural dimensions, the model highlights how individual agency interacts with systemic opportunity to shape well‐being and social participation in resettlement contexts. This integrated framework allows for a comprehensive examination of how individual characteristics, social resources, and structural contexts jointly influence cost‐related healthcare delays. While Andersen's model offers a structured approach to understanding who uses healthcare and why, Hynie's model situates these determinants within the broader ecology of refugee newcomer integration, emphasizing how social identity, cultural adaptation, and subjective belonging interact with institutional and socioeconomic environments.

Guided by this integrated framework (see Table 1), this study examines multilevel correlates of cost‐related healthcare delays among refugee newcomers in the United States. Using a data‐driven analytic approach, we first identified salient predictors across individual, structural, and contextual domains and then assessed how these factors were associated with delayed care.

**TABLE 1:** Integrated Andersen‐Hynie conceptual framework.

Integrated model dimension	Representative variables	Conceptual rationale
Predisposing/personal history	Sex, age, race and ethnicity, marital status, education, arrival‐cohort offset	Individual demographic and sociocultural characteristics shaping health beliefs, awareness, and initial predisposition toward healthcare use.
Enabling/social integration	Current English proficiency, household size, employment, driver's license, financial hardship, cash assistance, health insurance	Personal and community resources, institutional support, and social integration facilitating or constraining access to care.
Need/subjective integration	Self‐rated physical health and sadness frequency	Perceived and evaluated health status motivating health‐seeking behavior and influencing access decisions.
Contextual/socioeconomic context	Survey cycle, cultural similarity, initial resettlement region, community safety, discrimination, perceived welcome, trust in police, noncost healthcare barriers, bonding and bridging contact	Structural and environmental determinants representing integration context and regional or systemic influences on access.

## 2 Methods

### 2.1 Data Source and Study Sample

This study pooled publicly available, deidentified data from the Annual Survey of Refugees (ASR), a national cross‐sectional survey coordinated by the Office of Refugee Resettlement within the US Department of Health and Human Services to monitor refugee integration in the United States [19, 20, 21]. We used the 2020–2022 survey cycles, which cover arrival cohorts spanning fiscal years 2015–2021. Multilingual telephone interviews were conducted in 2021–2023. The ASR draws a stratified probability sample of households from administrative arrival records. Reported response rates were 17%, 19%, and 20% for the 2020, 2021, and 2022 cycles, respectively. The user guides identify difficulty locating sampled households as the main source of nonresponse and document response differences by arrival cohort and demographic characteristics. We included one designated household respondent per household in each cycle; household roster members did not contribute additional independent observations. Of 4532 respondent records, 48 lacked a valid outcome, seven outcome‐valid respondents were younger than 18, and 144 had unknown or refused age. The eligible sample comprised 4333 adults, of whom 3129 had complete data on all candidate variables. Annual household identifiers were combined with survey cycle and were not treated as longitudinal identifiers.

### 2.2 Measurement

A total of 26 theoretically informed candidate constructs spanning predisposing, enabling, need, and contextual domains were initially considered. Table 2 presents their response categories. Unknown, refused, and nonascertained responses were treated as missing, except for verified questionnaire skips.

**TABLE 2:** Characteristics of eligible respondents by reported cost‐related healthcare delay.

Characteristic	No delay (*n* = 3559)	Delay (*n* = 774)	Total (*N* = 4333)
Age in years	40.17 (12.59)	38.04 (10.96)	39.79 (12.34)
Sex			
Male	2013 (60.5)	424 (60.5)	2437 (60.5)
Female	1538 (39.5)	349 (39.5)	1887 (39.5)
Race and ethnicity			
White	1007 (20.5)	196 (23.0)	1203 (20.9)
Black	1060 (33.9)	193 (30.0)	1253 (33.2)
Asian	701 (25.7)	85 (14.3)	786 (23.6)
Middle Eastern/North African	309 (11.6)	99 (17.7)	408 (12.7)
Latino/Hispanic	332 (4.8)	170 (10.5)	502 (5.8)
Other	33 (0.8)	9 (1.7)	42 (1.0)
Multiple selections	71 (2.7)	15 (2.8)	86 (2.7)
Arrival‐cohort offset			
1 year	997 (8.3)	198 (7.2)	1195 (8.1)
2 years	580 (10.1)	140 (11.3)	720 (10.3)
3 years	600 (16.0)	129 (14.7)	729 (15.8)
4 years	575 (23.6)	126 (25.6)	701 (24.0)
5 years	772 (41.9)	173 (41.2)	945 (41.8)
Current English proficiency			
Very well	214 (8.0)	67 (10.5)	281 (8.4)
Well	1038 (33.3)	259 (39.3)	1297 (34.3)
Not well	1651 (41.1)	322 (36.7)	1973 (40.3)
Not at all	641 (17.7)	124 (13.5)	765 (16.9)
Education before US arrival			
No schooling	366 (13.0)	45 (7.9)	411 (12.1)
Primary or religious school	590 (18.7)	130 (17.8)	720 (18.5)
Lower secondary	638 (18.7)	106 (14.6)	744 (18.0)
Upper secondary or vocational	1302 (32.9)	286 (35.1)	1588 (33.3)
Some university or university degree	581 (14.7)	189 (22.3)	770 (16.0)
Other or camp classes	67 (2.0)	11 (2.4)	78 (2.1)
Marital status			
Married, spouse in household	1894 (53.0)	344 (43.5)	2238 (51.3)
Married, spouse elsewhere	325 (11.5)	56 (8.6)	381 (11.0)
Divorced, separated, or widowed	350 (11.3)	67 (10.2)	417 (11.1)
Never married or other single	909 (24.3)	291 (37.6)	1200 (26.6)
Household size			
1	654 (20.6)	158 (24.2)	812 (21.3)
2	406 (11.9)	91 (13.3)	497 (12.2)
3	509 (15.3)	124 (13.9)	633 (15.0)
4	609 (15.6)	122 (14.4)	731 (15.4)
5 or more	1380 (36.6)	278 (34.1)	1658 (36.1)
Employment status			
Employed	2297 (65.1)	546 (69.0)	2843 (65.8)
Unemployed	265 (8.1)	82 (11.5)	347 (8.7)
Not in labor force	923 (26.8)	138 (19.5)	1061 (25.5)
Difficulty meeting expenses			
No	3117 (86.6)	630 (81.2)	3747 (85.6)
Yes	395 (13.4)	133 (18.8)	528 (14.4)
Cash assistance			
No	2427 (67.1)	605 (77.8)	3032 (69.0)
Yes	1123 (32.9)	167 (22.2)	1290 (31.0)
Driver's license			
No	998 (23.3)	178 (16.0)	1176 (22.0)
Yes	2558 (76.7)	596 (84.0)	3154 (78.0)
Physical health score	2.90 (1.17)	3.19 (1.14)	2.95 (1.17)
Sadness frequency score	2.05 (1.19)	2.62 (1.26)	2.16 (1.22)
Insured months	10.50 (3.56)	8.11 (5.05)	10.07 (3.98)
Insurance type			
No insurance	244 (7.6)	164 (23.3)	408 (10.5)
Employment‐based	536 (20.0)	132 (18.5)	668 (19.8)
Medicaid/CHIP/RMA	2326 (60.2)	373 (42.9)	2699 (57.1)
Other, including Medicare	318 (12.1)	87 (15.2)	405 (12.7)
Any discrimination			
No	2784 (81.8)	438 (60.5)	3222 (78.0)
Yes	652 (18.2)	319 (39.5)	971 (22.0)
Perceived welcome			
Very welcome	2392 (69.3)	451 (58.7)	2843 (67.4)
Somewhat welcome	1005 (27.2)	272 (36.4)	1277 (28.8)
Somewhat or very unwelcome	101 (3.5)	42 (5.0)	143 (3.8)
Trust in police			
Not at all	77 (2.8)	43 (8.1)	120 (3.8)
Somewhat	719 (20.7)	196 (25.5)	915 (21.6)
A lot	2562 (76.5)	498 (66.5)	3060 (74.7)
Feels safe in community			
No	555 (15.0)	176 (22.0)	731 (16.3)
Yes	2803 (85.0)	568 (78.0)	3371 (83.7)
Neighbors sharing cultural background			
All or most	817 (21.9)	154 (18.5)	971 (21.3)
About half	525 (15.7)	108 (16.3)	633 (15.8)
Only a few	1464 (43.3)	314 (43.3)	1778 (43.3)
None	645 (19.1)	180 (21.9)	825 (19.6)
Bonding contact			
No	880 (24.8)	218 (30.1)	1098 (25.7)
Yes	2667 (75.2)	551 (69.9)	3218 (74.3)
Bridging contact			
No	2037 (57.1)	375 (48.9)	2412 (55.6)
Yes	1507 (42.9)	398 (51.1)	1905 (44.4)
Initial resettlement region			
Northeast	627 (15.7)	108 (12.7)	735 (15.2)
South	988 (29.2)	344 (39.8)	1332 (31.1)
Midwest	879 (28.2)	132 (22.1)	1011 (27.1)
West	1041 (26.9)	187 (25.3)	1228 (26.6)
Survey cycle			
2020	1169 (41.7)	285 (52.2)	1454 (43.6)
2021	1161 (34.6)	276 (31.0)	1437 (33.9)
2022	1229 (23.7)	213 (16.7)	1442 (22.5)
Any noncost healthcare barrier			
No	2145 (63.4)	289 (37.5)	2434 (58.7)
Yes	1386 (36.6)	483 (62.5)	1869 (41.3)

*Note:* Values are unweighted n (household‐weighted column %) or household‐weighted mean (standard deviation). Percentages use observed responses for each characteristic; item totals may be smaller than the column *N* because of missing responses. Higher physical health and sadness scores indicate poorer health and more frequent sadness.

Abbreviations: CHIP, Children's Health Insurance Program; RMA, Refugee Medical Assistance.

#### 2.2.1 Outcome Variable

The primary outcome was cost‐related delay in healthcare utilization, measured by respondents' report that they or anyone in their family had postponed or forgone needed medical care because of cost within the past 12 months. Responses were dichotomized to indicate any cost‐related delay (1) versus no delay (0).

#### 2.2.2 Predisposing Factors

Predisposing factors reflected demographic and resettlement‐related attributes shaping healthcare‐seeking tendencies. Race and ethnicity were measured using the ASR categories White, Black, Asian, Middle Eastern/North African, Latino/Hispanic, and Other, with a separate category for multiple selections (reference = White). Demographic information included sex (0 = male; 1 = female) and age, analyzed per 10 years with the released top code of 75 retained. Arrival‐cohort offset was calculated as nominal survey year minus arrival year and modeled categorically from 1 to 5 years; it does not measure exact residence duration at interview. Educational attainment represented schooling before US arrival (reference = no schooling). Marital status distinguished married respondents by whether their spouse lived in the household, alongside divorced/separated/widowed and never‐married/other‐single categories.

#### 2.2.3 Enabling Factors

Enabling factors captured material, institutional, and social resources that facilitate or constrain access to care. Current English language proficiency was measured using four categories, ranging from very well (1) to not at all (4). Household size counted household members, with five or more grouped together. Driver's license possession was coded as a binary indicator (1 = yes; 0 = no). Insurance‐related variables were central enabling measures. Insurance type distinguished employment‐based coverage, Medicaid/Children's Health Insurance Program (CHIP)/RMA, other coverage including Medicare, and no insurance. Insurance duration was measured as the number of months insured during the prior year (range: 0–12) and treated as continuous. Economic and institutional supports were captured through employment status (employed, unemployed, or not in the labor force) and binary indicators of difficulty meeting expenses and receipt of cash assistance.

#### 2.2.4 Need Factors

Need‐related variables represented perceived physical health and sadness frequency. Physical health was measured using a self‐rated health item ranging from excellent (1) to poor (5). The sadness item asked how often participants felt sad during the past 30 days and was reverse‐coded from none of the time (1) to all of the time (5), with higher values indicating more frequent sadness. Both five‐point scores were modeled as continuous variables.

#### 2.2.5 Contextual and Social Integration Factors

Contextual and social integration factors captured social environments, institutional experiences, and resettlement conditions shaping healthcare access. Discrimination combined eight setting‐specific items into an indicator of any discrimination. In 2020, a negative global screen led to skipped follow‐up items, which were coded as no discrimination; later cycles administered the setting‐specific items directly. Noncost healthcare barriers combined difficulty finding a provider, limited English ability, and lack of transportation (1 = any barrier; 0 = none). The composite excluded cost and an insurance item whose wording changed across cycles. Perceived welcome was modeled categorically as very welcome, somewhat welcome, or somewhat/very unwelcome, combining the last two responses because of sparse counts. Trust in local police was measured categorically (1 = not at all; 2 = somewhat; 3 = a lot). Sense of safety was coded as binary (1 = feels safe; 0 = does not feel safe). The proportion of neighbors sharing respondents' cultural background was modeled categorically (all or most, about half, only a few, or none). Initial resettlement region was coded as Northeast (reference), South, Midwest, or West. Bonding and bridging contact were measured using separate binary items on visits with friends or neighbors of similar and different cultural backgrounds.

### 2.3 Data Analysis

Analyses followed a multistage framework to identify correlates of cost‐related healthcare delays and examine racial and ethnic differences. Descriptive estimates used all eligible respondents with observed values for each characteristic. Counts were unweighted; percentages, means, and standard deviations used household population weights. Annual weights were divided by three to represent the pooled survey populations. Complete observations across the candidate set were used for variable selection and the primary multivariable model, ensuring a common analytical sample.

To address potential multicollinearity and improve model parsimony, survey‐weighted least absolute shrinkage and selection operator (LASSO) logistic regression was applied to the candidate variables [22]. Nominal variables were represented by indicators, and region and survey cycle were retained without penalization. Ten‐fold cross‐validation, stratified by cycle and outcome, evaluated weighted binomial deviance over 50 penalties; standardization used training‐fold weighted means and standard deviations. We selected the largest penalty within one standard error of the minimum deviance. If any indicator for a categorical construct was selected, all its levels were included in the subsequent unpenalized survey‐weighted logistic regression.

Adjusted associations are reported as average marginal effects (AMEs) in percentage points with 95% confidence intervals (CIs) [23, 24]. For categorical variables, AMEs were household‐weighted differences in predicted probabilities between each category and its reference, holding other characteristics at observed values. Continuous‐variable AMEs were weighted average probability derivatives. Each jackknife replicate refitted the model and recomputed these effects. The annual designs provided 35 replicates in 2020 and 60 in each subsequent cycle. Because the supplied 2021 population‐replicate series contained 406 additional missing cells across seven respondents, we constructed population‐scale replicates from household sample‐replicate weights, normalizing each to its annual full‐sample population total before analytical exclusions. This reproduced the supplied 2020 and 2022 population replicates to rounding. Pooled replicates perturbed one cycle at a time, retaining other cycles' main weights. Variances summed the within‐cycle jackknife contributions, centered on each cycle's mean replicate estimate, with the usual (*K* − 1)/*K* scaling for *K* replicates. Inference used 152 replicate degrees of freedom minus fitted slopes: 118 for the selected model and 97 for the full candidate model.

Sensitivity analyses compared weighted and unweighted refits on the same sample, minimum‐deviance selection, and full candidate adjustment. We also excluded 2021, restricted discrimination analyses to the directly administered grids, substituted the 2020 global screen, and replaced current English proficiency with proficiency at arrival. Alternative specifications treated health ratings categorically and grouped insured months as zero, one to 11, or 12. Transportation analyses replaced the barrier composite with its three components and included license possession under selected and full candidate adjustment. Among respondents selecting Latino/Hispanic, including multiple selections, descriptive analyses examined country of birth and grouped arrival‐cohort offsets.

Missing‐data sensitivity analyses used 50 independently initialized imputed datasets, each generated through 20 iterations of bootstrap classification and regression tree chained equations. Predictors included the outcome, candidate covariates, cycle, and log household weight. Observed donors were resampled within cycle, and missing values were drawn from terminal nodes of trees fitted with a minimum leaf size of 10. Observed values were preserved; neither the outcome nor age defining adult eligibility was imputed. Full candidate models were fitted to each completed dataset, and replicate variances were combined using Rubin's rules and Barnard‐Rubin degrees of freedom. Statistical analyses were conducted in R (version 4.3.3), using glmnet for LASSO, rpart for imputation, and custom routines for pooled jackknife inference; two‐tailed significance was set at *p* < 0.05.

## 3 Results

### 3.1 Descriptive Statistics

Among 4333 eligible respondents, 774 reported cost‐related healthcare delays for themselves or a family member, giving a weighted prevalence of 17.8% (95% CI, 16.4%–19.2%). Table 2 summarizes characteristics by reported delay. Respondents reporting delays had fewer insured months, poorer physical health, and more frequent sadness. Noncost healthcare barriers and discrimination were also more common among those reporting delays. Unadjusted delay prevalence was highest in the South (22.8%), compared with 15.0% in the Northeast, 14.6% in the Midwest, and 17.0% in the West. Complete cases had a prevalence of 18.4%, compared with 16.3% among respondents with incomplete covariates. Missingness was most frequent for insured months, trust in police, and community safety.

### 3.2 LASSO Regression Model

We applied LASSO logistic regression to identify a parsimonious set of correlates of cost‐related healthcare delay. Cross‐validation identified a minimum‐deviance penalty of 0.006594 and a one‐standard‐error penalty of 0.017691. The latter selected 13 constructs in addition to region and survey cycle, which were retained by design. Including all levels of selected categorical factors yielded 34 slopes and an intercept. Driver's license possession and current English proficiency were selected at the minimum‐deviance penalty but not under the one‐standard‐error criterion (Table A1).

Table 3 presents the final multivariable logistic regression model. Non‐cost healthcare barriers were associated with a 12.74‐percentage‐point higher probability of delay (95% CI, 8.76–16.71), and discrimination with a 9.40‐point increase (95% CI, 5.57–13.23). Each additional insured month was associated with a 1.06‐point decrease (95% CI, 0.58–1.53). Public coverage was associated with lower delay probability than no insurance. Black and Asian respondents had lower adjusted probabilities than White respondents; the Latino/Hispanic contrast was imprecise. Poorer physical health, more frequent sadness, and bridging contact were associated with higher probabilities. The South–Northeast contrast was 4.36 points (95% CI, −0.29 to 9.01), and the joint regional test did not clearly support adjusted differences, *F*(3, 118) = 1.56, *p* = 0.20.

**TABLE 3:** Survey‐weighted average marginal effects from the LASSO‐selected model of reported cost‐related healthcare delay.

Variable and contrast	AME, pp	95% CI, pp	*p*
Race and ethnicity: Black vs. White	−7.89	−12.65 to −3.14	0.001
Race and ethnicity: Asian vs. White	−9.43	−14.81 to −4.05	< 0.001
Race and ethnicity: Middle Eastern/North African vs. White	−1.55	−6.97 to 3.87	0.57
Race and ethnicity: Latino/Hispanic vs. White	3.14	−4.16 to 10.44	0.40
Race and ethnicity: Other vs. White	−12.07	−28.52 to 4.38	0.15
Race and ethnicity: Multiple selections vs. White	−3.73	−15.57 to 8.11	0.53
Education before US arrival: Primary or religious school vs. No schooling	5.75	0.05–11.45	0.048
Education before US arrival: Lower secondary vs. No schooling	1.79	−4.67 to 8.24	0.58
Education before US arrival: Upper secondary or vocational vs. No schooling	5.75	0.42–11.09	0.035
Education before US arrival: Some university or university degree vs. No schooling	7.92	1.34–14.51	0.019
Education before US arrival: Other or camp classes vs. No schooling	5.74	−3.99 to 15.48	0.24
Marital status: Married, spouse elsewhere vs. Married, spouse in household	0.02	−5.13 to 5.17	0.99
Marital status: Divorced, separated, or widowed vs. Married, spouse in household	0.49	−4.95 to 5.93	0.86
Marital status: Never married or other single vs. Married, spouse in household	4.81	0.77–8.85	0.020
Employment status: Unemployed vs. Employed	−1.91	−6.88 to 3.07	0.45
Employment status: Not in labor force vs. Employed	−5.83	−10.20 to −1.45	0.009
Physical health score per unit	1.91	0.32–3.50	0.019
Sadness frequency score per unit	3.91	2.61–5.21	< 0.001
Insured months per unit	−1.06	−1.53 to −0.58	< 0.001
Insurance type: Employment‐based vs. No insurance	−4.20	−10.82 to 2.42	0.21
Insurance type: Medicaid/CHIP/RMA vs. No insurance	−7.55	−14.25 to −0.85	0.028
Insurance type: Other, including Medicare vs. No insurance	−1.41	−7.66 to 4.84	0.66
Any discrimination: Yes vs. No	9.40	5.57–13.23	< 0.001
Perceived welcome: Somewhat welcome vs. Very welcome	2.75	−0.52 to 6.03	0.099
Perceived welcome: Somewhat or very unwelcome vs. Very welcome	−1.29	−8.06 to 5.49	0.71
Trust in police: Somewhat vs. Not at all	−11.57	−23.90 to 0.75	0.066
Trust in police: A lot vs. Not at all	−14.78	−27.24 to −2.32	0.020
Bridging contact: yes vs. no	4.04	0.80–7.28	0.015
Initial resettlement region: South vs. Northeast	4.36	−0.29 to 9.01	0.066
Initial resettlement region: Midwest vs. Northeast	0.57	−3.68 to 4.81	0.79
Initial resettlement region: West vs. Northeast	1.24	−3.58 to 6.06	0.61
Survey cycle: 2021 vs. 2020	−2.74	−7.14 to 1.67	0.22
Survey cycle: 2022 vs. 2020	−4.21	−8.20 to −0.23	0.039
Any noncost healthcare barrier: yes vs. no	12.74	8.76–16.71	< 0.001

*Note: N* = 3129; 591 reported delays.

Abbreviations: AME, average marginal effect; CHIP, Children's Health Insurance Program; CI, confidence interval; LASSO, least absolute shrinkage and selection operator; pp, percentage points; RMA, Refugee Medical Assistance.

The three principal associations persisted across the sensitivity analyses (Appendix Tables A2 and A6). Education contrasts in the selected model were attenuated with full candidate adjustment, and all education CIs included zero in the full candidate models using either complete or imputed covariates. Year‐round insurance was associated with less delay than no insured months, whereas the partial‐year contrast was uncertain (Appendix Table A3). Reported transportation difficulty remained associated with delay when examined separately from provider and language barriers; the license‐possession CI included zero under both adjustment sets (Appendix Table A4).

Among 521 respondents selecting Latino/Hispanic, weighted delay prevalence was 31.4% (95% CI, 25.6%–37.2%). Country‐specific estimates ranged from 21.3% for Colombia to 42.7% for Guatemala, but CIs were wide and overlapping. Grouped arrival‐cohort estimates did not establish a clear ordering (Appendix Table A5).

## 4 Discussion

This study examined cost‐related healthcare delays among a national sample of refugee newcomers in the United States. Findings highlight the importance of insurance coverage, practical barriers to obtaining care, and experiences of discrimination during resettlement. These associations are consistent with prior evidence that institutional conditions and the broader resettlement environment shape access to healthcare [25, 26, 27]. The integrated Andersen‐Hynie conceptual framework helped situate these findings across individual, social, and contextual domains. The LASSO‐based approach identified a parsimonious set of correlates, while the sensitivity analyses supported the principal patterns across alternative specifications.

Healthcare barriers and insurance duration were among the clearest correlates of delay. Difficulty finding a provider, communicating in English, or arranging transportation can make care difficult to obtain even when a person has insurance. This aligns with prior research documenting how procedural complexity and the work of navigating services contribute to unmet need [28, 29]. Studies of immigrant and refugee healthcare utilization similarly emphasize the importance of logistical support and health literacy [30, 31]. Longer insurance duration may indicate greater continuity in access; research among older refugees also links insurance coverage to healthcare use [11]. Maintaining coverage may help families establish a regular source of care and understand which services are available to them [32]. However, the ASR measure does not reveal why uninsured months occurred or whether coverage was interrupted repeatedly. Administrative churn and transitions between programs are plausible explanations [33], rather than mechanisms demonstrated by these data. The coexistence of coverage and noncost barriers also suggests that enrollment alone may be insufficient when families cannot navigate covered services [34, 35]. For families managing ongoing treatment, the consequences of these difficulties may accumulate across appointments, prescriptions, and referrals. A provider who accepts a particular plan may still be difficult to reach, and uncertainty about charges may discourage a visit even when some services are covered. These distinctions help explain why a cost‐related outcome can be associated with obstacles that are not themselves financial.

Social connections may also matter for healthcare access, although their role cannot be inferred from contact alone. Within Hynie's model, relationships form part of a wider process of integration alongside employment, housing, and institutional support [17, 18, 36]. Previous research describes how cross‐cultural ties can help newcomers navigate unfamiliar systems and obtain assistance [37, 38, 39]. In this analysis, however, bridging contact was associated with more reported delay. One possibility is that families facing unmet needs seek help beyond their immediate communities; another is that contact increases awareness of available care without making it affordable. The survey does not distinguish these explanations or measure the assistance exchanged. The finding therefore calls for closer examination of what social relationships provide, rather than treating the presence of cross‐cultural contact as evidence of effective support. Social capital concerns resources that can be accessed through relationships, whereas a report of visiting friends or neighbors establishes only that contact occurred. Understanding whether those ties offer information, transportation, financial help, or emotional support would make the connection to healthcare access more specific.

Relatedly, discrimination was associated with delayed care. This finding is consistent with research showing that racialized and stigmatizing encounters can discourage help‐seeking [40, 41, 42, 43] and contribute to avoidance of formal systems [44, 45, 46]. For refugee newcomers navigating unfamiliar institutions, such experiences may affect expectations of fair treatment and willingness to seek assistance. The ASR items cover discrimination across several settings, so the association cannot be attributed specifically to healthcare encounters. Nevertheless, the finding places financial concerns within a broader social context: the ability to seek care may depend partly on whether institutions are experienced as accessible and trustworthy.

Several findings also complicate conventional expectations about socioeconomic gradients in healthcare access [47]. Some education categories were associated with greater delay in the selected model, but these contrasts were not robust to full candidate adjustment or imputation. They should therefore be interpreted cautiously. Education before migration may not translate directly into resources after resettlement, particularly when refugees face labor market exclusion or barriers to credential recognition [48, 49]. Higher education might also affect expectations for care or awareness of unmet needs. In this context, a qualification earned before migration may carry little information about current employment quality or the financial resources available to a household. Assessing credential recognition and the match between prior training and current work would help determine whether educational resources translate into practical advantages in obtaining care.

The racial and ethnic findings likewise require attention to differences within broad survey categories. Black and Asian respondents had lower adjusted probabilities of delay than White respondents, whereas the Latino/Hispanic contrast remained uncertain. Prior immigrant health research describes distinct experiences of stigma, exclusion, and institutional vulnerability across racialized groups [50, 51, 52]. Within the Latino/Hispanic population, however, national origin alone may conceal differences in preferred language, Indigenous identity, migration pathway, and local support. Newly resettled refugees may receive assistance unavailable to immigrants admitted through other pathways, yet still have limited familiarity with services. Longer residence may improve navigation while leaving people vulnerable when coverage ends or health needs increase. Those facing limited language support, interrupted coverage, and substantial treatment needs may consequently require additional assistance, regardless of arrival cohort. The subgroup estimates do not establish that one national‐origin group or arrival cohort is consistently most disadvantaged. Language categories require similar care: identifying a respondent as Latino/Hispanic does not establish a preference for Spanish or access to an interpreter in the language used at home. Service planning should therefore distinguish the resources available to a particular family from assumptions based on a broad ethnic label. This is especially relevant when formal eligibility for assistance and the ability to navigate that assistance differ.

Respondents reporting poorer physical health or more frequent sadness were also more likely to report delays. Within Andersen's model, greater need would generally be expected to encourage healthcare use [15, 16], but obtaining care still depends on enabling resources. People who require repeated visits or ongoing treatment may have more opportunities to encounter costs and practical obstacles. This helps explain why coverage and navigation support remain relevant even when the need for care is readily recognized.

The higher unadjusted delay rate in the South was not clearly supported after adjustment. Differences in measured population characteristics may contribute to the descriptive gradient, but these results do not identify the roles of Medicaid policy, safety‐net capacity, or service availability. Initial resettlement region is too broad to represent the policies and providers encountered by families who may have moved. The regional findings therefore motivate more geographically detailed research rather than an explanation based on unmeasured state or local mechanisms.

## 5 Limitations

Several limitations should be noted when interpreting the findings. First, the study relies on cross‐sectional data, which limits causal inference. The family‐level outcome and predominantly respondent‐level covariates may refer to different household members. While the ASR is a comprehensive national dataset on refugee experiences, it is not specifically designed for health research. Many psychosocial constructs were measured using brief self‐report items, limiting the precision with which mechanisms can be interpreted. The data do not distinguish delayed from forgone care or identify the service or family member affected. Driver's license possession does not establish access to a vehicle, public transit, or transport through family or agencies and may reflect administrative integration. Racial and ethnic self‐identification may also vary across cultural and linguistic contexts, and sparse country‐specific samples limit subgroup comparisons. The sadness item is a measure of symptom frequency, not a diagnostic assessment, and does not establish a need for a particular treatment. Recall and differences in expectations of care may also affect whether respondents identify an unmet need and attribute it to cost.

Analytically, the annual samples were treated as independent because repeat participation could not be verified across cycles. Survey weights account for selection probabilities and measured nonresponse adjustments but cannot eliminate bias related to unmeasured participation factors. LASSO selection can be model‐dependent, particularly for correlated predictors, and the reported intervals condition on the selected specification without incorporating selection uncertainty. Complete‐case analysis may introduce bias if missingness is systematically related to healthcare access; the imputation sensitivity assumes that missingness is explained by the included predictors.

## 6 Health System and Policy Implications

The findings support efforts to improve coverage continuity while reducing the practical burden of obtaining care. Assistance with transitions from RMA to Medicaid or marketplace coverage should be coordinated with provider navigation, so that families can both maintain coverage and reach appropriate services. Clear explanations of benefits and expected charges may reduce uncertainty during care seeking. The transportation findings also support asking directly about travel needs rather than relying on license possession to identify who needs assistance.

Changes in coverage rules and resettlement policy have also altered the setting in which these services operate. The observation period overlapped the 2022 extension of RMA to 12 months and the beginning of Medicaid unwinding in April 2023 [53, 54]. RMA was subsequently reduced to 4 months in 2025 and expanded to 8 months in July 2026 for qualifying individuals [53]. The 2025 suspension of refugee admissions, followed by a May 2026 determination raising the fiscal‐year ceiling to 17,500 with additional places allocated to Afrikaners from South Africa, also changed the scale and composition of planned admissions [55, 56]. Restrictions on federal Medicaid and CHIP financing for some noncitizen categories are scheduled to begin in October 2026, subject to statutory exceptions [57]. Such changes can alter families' coverage options and the demands on local services. Our estimates provide a historical reference for assessing these transitions; they do not measure the effects of the later policies or changes in safety‐net capacity.

The association with discrimination further supports attention to institutional practices alongside enrollment and navigation. Addressing these concerns requires more than individual cultural competence training [58]. Health systems can strengthen procedures for responding to discriminatory treatment and work with community organizations to identify obstacles that are missed by routine eligibility screening. These approaches should be evaluated through timely receipt of needed care and patients' experiences of affordability and treatment. Linking refugee survey data with local service and policy information would help identify where these efforts are most needed and whether they reduce disparities. Partnerships with refugee‐serving organizations could support this work by bringing patient experiences into service planning and identifying where referrals fail to lead to completed care.

## 7 Conclusion

Cost‐related healthcare delay among refugee newcomers was consistently associated with shorter insurance coverage, practical barriers to care, and discrimination. These findings place affordability within the institutional and social conditions of resettlement and support closer coordination between health systems and resettlement agencies. Assistance with coverage transitions should be connected to the services families need to find providers, communicate their needs, and attend appointments. Addressing discriminatory treatment is also integral to making those services accessible. As eligibility rules and resettlement policies change, their consequences should be assessed through the care families actually receive. Timely, affordable care should be a measure of whether resettlement support is meeting refugee families' needs.

## Author Contributions

**Hyojin Im:** conceptualization, data curation, formal analysis, investigation, writing original draft, writing review and editing. **Isok Kim:** conceptualization, investigation, writing original draft, writing review and editing. **Wooksoo Kim:** conceptualization, investigation, writing original draft, writing review and editing. All authors have read and agreed to the published version of the manuscript.

## Funding

The authors have nothing to report.

## Ethics Statement

This study involved secondary analysis of de‐identified data from the 2020 Annual Survey of Refugees (ASR), a publicly available dataset. As such, it does not constitute human subjects research as defined by federal regulations and did not require formal ethics approval. The Virginia Commonwealth University Institutional Review Board (VCU IRB) reviewed the study protocol and determined it to be exempt, with the exemption number HM20029828. All original data collection procedures for the ASR were conducted in compliance with ethical standards, including obtaining informed consent from participants. The present secondary analysis was conducted in accordance with the principles of the Declaration of Helsinki.

## Consent

Informed consent was obtained from participants.

## Conflicts of Interest

The authors declare no conflicts of interest.

## Supporting information


**Table A1.** Candidate variables and LASSO selection.
**Table A2**. Sensitivity of the principal associations.
**Table A3**. Insurance duration modeled categorically.
**Table A4**. Transportation and other access barriers.
**Table A5**. Delay among Latino/Hispanic respondents.
**Table A6**. Full candidate models with complete and imputed covariates.

## Data Availability

This study used de‐identified public‐use microdata from the 2020–2022 Annual Survey of Refugees (ASR), conducted by the Urban Institute and SSRS on behalf of the U.S. Department of Health and Human Services’ Office of Refugee Resettlement. The datasets and accompanying documentation are publicly available through the Inter‐university Consortium for Political and Social Research (ICPSR), with links to each survey cycle provided at https://www.urban.org/projects/annual‐survey‐refugees/researchers.
