# Supplementary material for: Structural Determinants of Cost‐Related Healthcare Delay Among Refugee Newcomers in the United States: Evidence From the Annual Survey of Refugees, 2020–2022
Source: Health Serv Res. 2026 Nov 13;61(6):e70184. doi: 10.1111/1475-6773.70184 (PMC13768149; doi:10.1111/1475-6773.70184)
Supplement: Supplementary file 1 [file HESR-61-e70184-s001.docx]

Supplementary analyses

LASSO denotes least absolute shrinkage and selection operator. Regression estimates are average marginal effects (AMEs) in percentage points (pp); parentheses contain 95% confidence intervals (CIs).

# Table A1 Candidate variables and LASSO selection

| **Candidate variable** | **Minimum deviance** | **One standard error** |
| --- | --- | --- |
| Age in years | Yes | No |
| Sex | No | No |
| Race and ethnicity | Yes | Yes |
| Arrival-cohort offset | No | No |
| Current English proficiency | Yes | No |
| Education before US arrival | Yes | Yes |
| Marital status | Yes | Yes |
| Household size | No | No |
| Employment status | Yes | Yes |
| Difficulty meeting expenses | Yes | No |
| Cash assistance | Yes | No |
| Driver’s license | Yes | No |
| Physical health score | Yes | Yes |
| Sadness frequency score | Yes | Yes |
| Insured months | Yes | Yes |
| Insurance type | Yes | Yes |
| Any discrimination | Yes | Yes |
| Perceived welcome | Yes | Yes |
| Trust in police | Yes | Yes |
| Feels safe in community | Yes | No |
| Neighbors sharing cultural background | No | No |
| Bonding contact | No | No |
| Bridging contact | Yes | Yes |
| Initial resettlement region | Retained | Retained |
| Survey cycle | Retained | Retained |
| Any non-cost healthcare barrier | Yes | Yes |

# Table A2 Sensitivity of the principal associations

| **Specification** | **N** | **Non-cost barriers** | **Discrimination** | **Insured month** |
| --- | --- | --- | --- | --- |
| Unweighted selected model | 3,129 | 11.09 | 10.01 | −1.04 |
| Minimum-deviance selection | 3,129 | 13.35 (9.38, 17.32) | 8.49 (4.57, 12.41) | −1.04 (−1.52, −0.56) |
| Exclude 2021 | 2,071 | 15.23 (9.93, 20.53) | 10.56 (5.58, 15.55) | −0.99 (−1.65, −0.33) |
| Direct discrimination grids, 2021-2022 | 2,122 | 8.58 (4.41, 12.74) | 9.61 (5.10, 14.12) | −0.96 (−1.41, −0.51) |
| 2020 discrimination screen substituted | 3,128 | 12.74 (8.76, 16.71) | 9.44 (5.61, 13.27) | −1.06 (−1.53, −0.58) |
| Categorical health ratings | 3,129 | 12.51 (8.57, 16.46) | 9.56 (5.71, 13.42) | −1.02 (−1.51, −0.54) |
| English at arrival, full model | 3,137 | 13.38 (9.32, 17.44) | 8.15 (4.30, 12.01) | −1.04 (−1.52, −0.55) |

NOTE: The unweighted row reports point estimates only. Primary estimates appear in Table 3; full candidate and imputed models appear in Table A6.

# Table A3 Insurance duration modeled categorically

| **Coverage contrast** | **AME, pp** | **95% CI, pp** |
| --- | --- | --- |
| 1-11 months vs 0 months | −1.24 | −8.79, 6.30 |
| 12 months vs 0 months | −12.59 | −20.07, −5.11 |

NOTE: N = 3,129. Adjustment includes the selected covariates. Joint test: F(2, 117) = 11.70, P < .001.

# Table A4 Transportation and other access barriers

| **Contrast** | **Selected adjustment** | **Full candidate adjustment** |
| --- | --- | --- |
| Driver’s license (yes vs no) | 3.10 (−1.03, 7.24) | 2.32 (−1.96, 6.60) |
| Difficulty finding a provider (yes vs no) | 18.24 (12.73, 23.74) | 18.52 (12.83, 24.21) |
| Limited English as a barrier (yes vs no) | 1.64 (−2.04, 5.32) | 2.02 (−1.69, 5.72) |
| Lack of transportation (yes vs no) | 8.48 (2.61, 14.34) | 8.69 (2.71, 14.66) |

NOTE: N = 3,123; 590 reported delays.

# Table A5 Delay among Latino/Hispanic respondents

| **Group** | **n** | **Delays, n** | **Weighted %** | **95% CI** |
| --- | --- | --- | --- | --- |
| All respondents | 521 | 174 | 31.4 | 25.6, 37.2 |
| El Salvador | 309 | 106 | 33.3 | 26.1, 40.5 |
| Colombia | 77 | 20 | 21.3 | 9.2, 33.3 |
| Other | 68 | 26 | 35.6 | 15.0, 56.2 |
| Guatemala | 65 | 22 | 42.7 | 24.7, 60.6 |
| Arrival offset 1 year | 166 | 50 | 26.6 | 17.7, 35.4 |
| Arrival offset 2-3 years | 200 | 70 | 33.3 | 25.0, 41.5 |
| Arrival offset 4-5 years | 151 | 51 | 30.7 | 22.1, 39.3 |

NOTE: Country categories with fewer than 10 respondents are suppressed (two respondents). Four respondents lack arrival-year data. Other is the survey response category.

# Table A6 Full candidate models with complete and imputed covariates

| **Variable or category** | **Complete cases N = 3,129** | **Multiple imputation N = 4,333** |
| --- | --- | --- |
| Age in years (per 10 years) | −1.12 (−2.94, 0.70) | −1.42 (−2.90, 0.05) |
| Sex (female vs male) | 0.04 (−3.69, 3.77) | −0.18 (−3.47, 3.11) |
| **Race and ethnicity (reference: White)** |  |  |
| Black | −7.91 (−12.76, −3.06) | −6.65 (−10.62, −2.68) |
| Asian | −9.64 (−15.16, −4.12) | −10.20 (−14.74, −5.65) |
| Middle Eastern/North African | −2.37 (−8.09, 3.36) | 0.14 (−5.17, 5.44) |
| Latino/Hispanic | 4.84 (−3.44, 13.12) | 4.48 (−2.13, 11.09) |
| Other | −11.44 (−29.66, 6.78) | 1.99 (−11.67, 15.65) |
| Multiple selections | −3.76 (−16.19, 8.66) | −5.26 (−15.64, 5.11) |
| **Arrival-cohort offset (reference: 1 year)** |  |  |
| 2 years | 0.95 (−3.60, 5.50) | 1.74 (−1.68, 5.15) |
| 3 years | −0.41 (−4.81, 4.00) | −0.56 (−4.13, 3.02) |
| 4 years | 1.89 (−3.22, 6.99) | 2.12 (−1.81, 6.05) |
| 5 years | 0.71 (−3.58, 5.00) | 1.30 (−2.13, 4.74) |
| **Current English proficiency (reference: Very well)** |  |  |
| Well | −3.12 (−9.18, 2.93) | −3.39 (−9.18, 2.41) |
| Not well | −4.56 (−10.79, 1.66) | −4.86 (−11.31, 1.59) |
| Not at all | −5.11 (−13.17, 2.95) | −6.24 (−13.17, 0.69) |
| **Education before US arrival (reference: No schooling)** |  |  |
| Primary or religious school | 5.46 (−1.01, 11.92) | 3.44 (−1.68, 8.56) |
| Lower secondary | 1.08 (−5.87, 8.04) | −0.46 (−5.52, 4.61) |
| Upper secondary or vocational | 4.51 (−1.73, 10.74) | 2.24 (−2.96, 7.45) |
| Some university or university degree | 6.26 (−1.44, 13.96) | 4.34 (−2.15, 10.83) |
| Other or camp classes | 5.97 (−4.74, 16.69) | 5.49 (−4.66, 15.63) |
| **Marital status (reference: Married, spouse in household)** |  |  |
| Married, spouse elsewhere | −0.78 (−5.83, 4.27) | −1.14 (−5.56, 3.29) |
| Divorced, separated, or widowed | 1.06 (−5.28, 7.40) | 0.49 (−5.03, 6.02) |
| Never married or other single | 3.23 (−1.12, 7.57) | 3.05 (−0.67, 6.76) |
| **Household size (reference: 1)** |  |  |
| 2 | −2.36 (−7.60, 2.87) | 1.38 (−3.48, 6.24) |
| 3 | −1.71 (−7.01, 3.60) | −0.20 (−4.73, 4.32) |
| 4 | −0.50 (−7.02, 6.03) | −0.33 (−5.38, 4.72) |
| 5 or more | −1.48 (−6.40, 3.45) | 1.20 (−2.87, 5.26) |
| **Employment status (reference: Employed)** |  |  |
| Unemployed | −2.03 (−7.20, 3.13) | −0.84 (−5.29, 3.61) |
| Not in labor force | −5.23 (−9.93, −0.53) | −3.67 (−7.33, −0.01) |
| Difficulty meeting expenses (yes vs no) | 2.34 (−2.56, 7.24) | 2.91 (−1.01, 6.83) |
| Cash assistance (yes vs no) | −0.60 (−5.29, 4.09) | −2.57 (−6.19, 1.05) |
| Driver’s license (yes vs no) | 2.28 (−2.11, 6.67) | 3.16 (−0.55, 6.87) |
| Physical health score (per unit) | 2.49 (0.75, 4.23) | 3.26 (1.62, 4.90) |
| Sadness frequency score (per unit) | 3.77 (2.49, 5.05) | 3.43 (2.38, 4.47) |
| Insured months (per month) | −1.02 (−1.52, −0.52) | −1.01 (−1.45, −0.57) |
| **Insurance type (reference: No insurance)** |  |  |
| Employment-based | −4.62 (−11.55, 2.31) | −5.80 (−11.70, 0.09) |
| Medicaid/CHIP/RMA | −7.47 (−14.46, −0.49) | −6.92 (−13.00, −0.83) |
| Other, including Medicare | −1.28 (−8.21, 5.64) | −2.97 (−8.83, 2.88) |
| Any discrimination (yes vs no) | 8.35 (4.47, 12.23) | 8.41 (4.82, 11.99) |
| **Perceived welcome (reference: Very welcome)** |  |  |
| Somewhat welcome | 2.60 (−0.91, 6.11) | 1.33 (−1.72, 4.39) |
| Somewhat or very unwelcome | −1.57 (−8.78, 5.64) | −1.28 (−7.43, 4.86) |
| **Trust in police (reference: Not at all)** |  |  |
| Somewhat | −10.75 (−23.15, 1.65) | −7.64 (−16.52, 1.24) |
| A lot | −13.42 (−26.03, −0.81) | −9.04 (−18.05, −0.04) |
| Feels safe in community (yes vs no) | −1.05 (−5.32, 3.22) | −1.86 (−5.42, 1.70) |
| **Neighbors sharing cultural background (reference: All or most)** |  |  |
| About half | 3.26 (−2.36, 8.89) | 1.52 (−2.98, 6.01) |
| Only a few | 1.60 (−2.89, 6.10) | 1.08 (−2.87, 5.02) |
| None | 2.58 (−2.36, 7.53) | 0.58 (−3.14, 4.29) |
| Bonding contact (yes vs no) | −0.89 (−4.81, 3.02) | −3.35 (−6.48, −0.21) |
| Bridging contact (yes vs no) | 4.07 (0.77, 7.38) | 4.10 (1.33, 6.86) |
| **Initial resettlement region (reference: Northeast)** |  |  |
| South | 4.01 (−0.88, 8.90) | 3.64 (−0.82, 8.11) |
| Midwest | 0.51 (−3.90, 4.92) | 0.80 (−2.52, 4.12) |
| West | 0.96 (−4.08, 6.00) | 0.79 (−3.48, 5.06) |
| **Survey cycle (reference: 2020)** |  |  |
| 2021 | −2.63 (−7.15, 1.90) | −2.57 (−6.13, 0.99) |
| 2022 | −4.14 (−8.03, −0.24) | −4.69 (−7.99, −1.39) |
| Any non-cost healthcare barrier (yes vs no) | 13.50 (9.43, 17.57) | 12.03 (8.73, 15.34) |

NOTE: CHIP, Children’s Health Insurance Program; RMA, Refugee Medical Assistance.
